# Family member’s perceived impact of anorexia nervosa and bulimia nervosa on family dynamics: a qualitative systematic review and meta-synthesis

**DOI:** 10.1590/0102-311XEN181525

**Published:** 2026-05-01

**Authors:** Thaís Shirane, Diogo Sussumu Okasawara, Érika Arantes Oliveira-Cardoso, Manoel Antônio dos Santos

**Affiliations:** 1 Faculdade de Filosofia, Ciências e Letras de Ribeirão Preto, Universidade de São Paulo, Ribeirão Preto, Brasil.

**Keywords:** Eating Disorder, Family Dynamics, Mental Health Services, Systematic Review, Transtornos da Alimentação, Dinâmica Familiar, Serviços de Saúde Mental, Revisão Sistemática, Trastorno Alimentario, Dinámica Familiar, Servicios de Salud Mental, Revisión Sistemática

## Abstract

Eating disorders configure a growing and complex public health issue which hinders implementing targeted policies. Global health policies emphasize tailored interventions for reducing eating disorders harms among vulnerable people. This systematic literature review and meta-synthesis interprets the synthesized findings from primary qualitative studies on the experience of family living with the diagnosis of anorexia nervosa and bulimia nervosa. The study was conducted based on the SPIDER search strategy and PRISMA guidelines on nine databases: Academic Search Premier, CINAHL, LILACS, MEDLINE, PubMed, PsycINFO, SocINDEX, Scopus, and Web of Science. Two independent reviewers performed the article screening and selection processes. Of the 2,269 studies initially identified, 28 met the inclusion criteria and were selected. Four descriptive themes emerged: (1) Anorexia nervosa/bulimia nervosa within the family environment: meanings attributed to the symptoms; (2) Anorexia nervosa/bulimia nervosa dominance within the family system: care that also harms; (3) Challenges in accessing care: individual and social barriers; and (4) Pathways to coping: supporting the whole family - a public health issue. The study shows that family dynamics and the health-illness process are closely linked, forming a cyclical, mutually reinforcing relationship that highlights the need for a more comprehensive approach to supporting the whole family. These findings corroborate the need for global policies that emphasize tailored interventions to reduce eating disorders harms and suffering within families.

## Abstract (pt)

Transtornos alimentares, uma questão crescente e complexa de Saúde Pública, dificultam a implementação de políticas direcionadas. As políticas globais de saúde enfatizam a necessidade de intervenções personalizadas para reduzir os danos causados por esses transtornos entre pessoas vulneráveis. Esta revisão sistemática da literatura e metassíntese buscou interpretar os achados de estudos qualitativos primários sobre a experiência de famílias vivendo com um diagnóstico de anorexia nervosa e bulimia nervosa. Este estudo foi conduzido com base na estratégia de busca SPIDER e nas diretrizes PRISMA em nove bancos de dados: Academic Search Premier, CINAHL, LILACS, MEDLINE, PubMed, PsycINFO, SocINDEX, Scopus e Web of Science. No todo, dois revisores independentes conduziram o processo metodológico de triagem e seleção dos artigos. Um total de 2.269 estudos foram achados; 28 dos quais (atendendo aos critérios de inclusão) foram selecionados. No todo, quatro temas descritivos foram apresentados: (1) Anorexia nervosa/bulimia nervosa dentro do ambiente familiar: os significados atribuídos aos sintomas; (2) A predominância da anorexia nervosa/bulimia nervosa dentro do sistema familiar: cuidado que também causa danos; (3) Desafios no acesso ao cuidado: barreiras individuais e sociais; e (4) Caminhos para lidar com o enfrentamento: apoio à toda a família - uma questão de Saúde Pública. O estudo mostra que a dinâmica familiar e o processo de saúde e doença estão intimamente ligados, formando uma relação cíclica e mutuamente reforçadora que destaca a necessidade de uma abordagem mais abrangente para apoiar toda a família. Os dados corroboram a necessidade de políticas globais que enfatizem intervenções personalizadas para reduzir os danos e o sofrimento das famílias afetadas pelos transtornos alimentares.

## Abstract (es)

Los trastornos alimentarios son un problema de salud pública cada vez más grave y complejo, lo que dificulta la aplicación de políticas específicas. Las políticas sanitarias mundiales enfatizan intervenciones personalizadas para reducir los daños causados por los trastornos alimentarios entre las personas vulnerables. Este artículo presenta una revisión sistemática de la literatura y una meta-síntesis para interpretar los resultados sintetizados de estudios cualitativos primarios sobre la experiencia de las familias que conviven con el diagnóstico de anorexia nerviosa y bulimia nerviosa. El estudio se llevó a cabo basándose en la estrategia de búsqueda SPIDER y las directrices PRISMA en nueve bases de datos: Academic Search Premier, CINAHL, LILACS, MEDLINE, PubMed, PsycINFO, SocINDEX, Scopus y Web of Science. Dos revisores independientes llevaron a cabo el proceso metodológico de selección y cribado de artículos. Se recuperaron inicialmente un total de 2.269 estudios; 28 artículos cumplían los criterios de inclusión y fueron seleccionados. Se elaboraron cuatro temas descriptivos: (1) Anorexia nerviosa/bulimia nerviosa en el entorno familiar: los significados atribuidos a los síntomas; (2) El predominio de la anorexia nerviosa/bulimia nerviosa en el sistema familiar: cuidados que también causan daño; (3) Retos para acceder a la atención: barreras individuales y sociales; (4) Vías para afrontar la situación: apoyar a toda la familia -una cuestión de salud pública. El estudio muestra que la dinámica familiar y el proceso de salud-enfermedad están estrechamente relacionados, formando una relación cíclica que se refuerza mutuamente y que pone de relieve la necesidad de un enfoque más integral para apoyar a toda la familia. Los datos corroboran la necesidad de políticas globales que enfaztizan intervenciones personalizadas para reducir los daños y el sufrimiento de las familias afectadas por los trastornos alimentarios.

## Introduction

Eating disorders have showed a significantly increased incidence in recent decades ^1^. Anorexia nervosa ^2^ and bulimia nervosa ^3^, two potentially life-threatening and persistent disorders, have the highest mortality rate out of any psychiatric complication ^4^. Other common eating disorders include binge eating disorder, avoidant restrictive food intake disorder (ARFID) and other non-specified feeding and eating disorder ^5^.

Affecting the health of large population groups worldwide, these conditions require both collective and governmental action to be effectively addressed. Such issues are not only medical but also social, environmental, political, and economic, as they influence overall well-being ^2^ ^,^ ^6^ ^,^ ^7^. This phenomenon represents a growing and complex public health concern, further intensified by inconsistencies in healthcare systems and the pervasive influence of social determinants of health such as poverty, inequality, and limited access to care.

Addressing these challenges requires global health policies involving governments, international organizations, nongovernmental organizations, and public-private partnerships to promote equitable access to care. Developing international health strategies shaped by all these stakeholders is essential to address transnational health challenges and promote equity in access to healthcare ^5^ ^,^ ^6^.

Failures in management and coordination across care levels, along with the unpreparedness of primary care to identify and manage eating disorders and the under-resourced, overburdened specialist services unable to accommodate high referral volumes, create significant weaknesses in the healthcare system ^6^. Thus, treatment becomes a challenging and long-term procedure for families and patients ^2^ ^,^ ^6^ ^,^ ^7^.

Family plays a critical role in eating disorders by taking an active position in the care and rehabilitation process of individuals with mental disorders. Psychosocial care marks a significant shift from the hospital-centered model which often blamed or excluded families from the care process ^8^. Mental health policies is one of the cornerstones for building a more supportive, welcoming, resilient, and just society ^9^.

The family of patients with eating disorder is no longer seen as peripheral but as an active agent in therapeutic outcomes, assuming a key role both in providing emotional and practical support to patients and in promoting a broader social support network. Moreover, family involvement is crucial for preventing relapse, supporting patients during crises, and assisting in patients’ reintegration into the community ^10^ ^,^ ^11^. Family education programs and psychosocial support are common practices aimed at informing family members on how to cope more effectively with eating disorders ^12^.

In cases of a parent with an eating disorder, their parenting capacities may be compromised. Studies indicate that mothers with eating disorder often face challenges during pregnancy and parenthood demands ^13^ ^,^ ^14^. Understanding the complexities of parenting while living with an eating disorder is essential to develop effective strategies supporting both affected parents and their children.

When a child develops an eating disorder, however, parental roles and the overall family dynamic may be profoundly disrupted. The ripple effects within the family system can be substantial ^15^ ^,^ ^16^, especially as symptoms often emerge during critical periods of child development and family formation.

Eating disorders’ chronic nature and high comorbidity with other mental health conditions suggest that their impact extends beyond individual symptoms, influencing interpersonal relationships and contributing to caregiver distress ^17^ ^,^ ^18^ ^,^ ^19^. However, the lack of clear evidence regarding these effects presents a significant challenge to developing comprehensive treatment approaches that address both individual recovery and family well-being ^20^.

While extensive research has documented the influence of family relations on eating disorders development ^15^ ^,^ ^21^ ^,^ ^22^ ^,^ ^23^ ^,^ ^24^ ^,^ ^25^ ^,^ ^26^, there remains a critical gap in our understanding of how these conditions affect the broader family system. Despite the well-established bidirectional relationship between family dynamics and mental health outcomes ^27^ ^,^ ^28^ ^,^ ^29^, eating disorder-specific effects on family functioning and parent-child interactions remain understudied.

Without a thorough understanding of how eating disorders influence family functioning and parent-child relationships, clinicians face challenges in tailoring interventions that effectively support both the affected individual and their family members ^10^ ^,^ ^30^. This research gap is particularly concerning given the increasingly recognized importance of family-based interventions in treatment ^10^ ^,^ ^31^ ^,^ ^32^.

Given this context, we formulated the following research question: What are the multifaceted impacts of eating disorders on family functioning? By investigating these interconnections, we contribute to developing more effective family-centered interventions and support strategies in family contexts affected by eating disorders. Moreover, a meta-synthesis design enables the systematic retrieval, expansion, and reinterpretation of knowledge beyond initial descriptions, creating more than just a sum of sources and establishing a fundamental connection between the results while identifying gaps and issues that require further exploration and debate ^33^.

## Methods

### Design

The systematic review and meta-synthesis were conducted following ten steps ^34^ ^,^ ^35^ ^,^ ^36^ ^,^ ^37^ ^,^ ^38^ ^,^ ^39^: (1) Development of the research question based on the SPIDER strategy; (2) Definition of selection and exclusion criteria, and choice of appropriate databases for the research area; (3) Elaboration of the search strategy based on specific descriptors for each database; (4) Database searches with validation by another researcher who independently evaluated the information; (5) Screening and selection from titles and abstracts and reviewing the results with a second independent researcher and using the Rayyan tool (https://www.rayyan.ai/); (6) Calculation of the Cohen’s kappa index of inter-rater agreement ^40^; (7) Full-text reading and final selection of studies; (8) Qualitative analysis of the methodological procedures of the reviewed studies based on the *Critical Appraisal Skills Program* (CASP) ^41^; (9) Coding the results of selected articles using the QDA Miner Lite 9.0 program (https://provalisresearch.com/); and (10) Description and analysis of the material.

This study was registered on the International Prospective Register of Systematic Reviews (PROSPERO; protocol CRD42024615832) ^42^. To report the essential elements that should be included in a qualitative evidence synthesis, the *Enhancing Transparency in Reporting the Synthesis of Qualitative Research* (ENTREQ) guide was used ^43^.

All stages were performed by two reviewers with prior expertise in meta-synthesis and qualitative research methodology, which facilitates new interpretations of an experience, offering a fresh conceptual understanding of the synthesized results, surpassing previous findings and developing new understandings. Ethics approval was not required, as only published studies were reviewed.

### Research question, eligibility criteria and research strategy

Given the research question formulated (What qualitative evidence is available in the scientific literature regarding family members’ perceived impact of anorexia and bulimia nervosa on family dynamics?) evidence was retrieved using the SPIDER⁠ strategy ([S] sample; [PI] phenomenon of interest; [D] study design; [E] evaluation; [R] research type), as it is particularly suited to qualitative research methods and ensures greater study rigor.

Eligibility criteria included: (i) primary qualitative studies originally published in English, Portuguese or Spanish; (ii) studies consistent with the research question developed using the SPIDER strategy; and (iii) inclusion of family members’ perceptions (parents or siblings) in the results. Exclusion criteria consisted of: (i) quantitative, mixed, secondary and literature review; (ii) gray literature like theses, dissertations, monographs, books or chapters; and (iii) letters to the editor, editorials, commentaries, opinion articles, and abstracts.

Databases were selected by their relevance to the field of knowledge, and by their national and international scope. SPIDER search strategy was defined by combining the descriptors of each acronym, choosing the appropriate descriptors for each database. Bibliographic search used the Boolean operators OR between descriptors of the same acronym and AND between each one of them, using a single search strategy adapted to each database that includes: (S1 OR S2...) AND (Pi1 OR Pi2...) AND (D1 OR D2...) AND (E1 OR E2...) AND (R1 OR R2...). “Advanced search” tool was used in the databases. Supplementary Material (Box S1; https://cadernos.ensp.fiocruz.br/static//arquivo/suppl-e00181525_1729.pdf) presents a track of descriptors compiled from Health Science Descriptors/Medical Subject Headings (DeCS/MeSH).

### Paper retrieval and study selection

Two independent reviewers conducted the search in September 2024 across nine databases (Academic Search Premier, CINAHL, LILACS, MEDLINE, PubMed, PsycInfo, SocINDEX, Scopus and Web of Science), initially retrieving 2,269 articles.

Article selection was refined using Rayyan software for systematic reviews ^44^ which improves screening efficiency and accuracy, ensuring transparency and reliability ^45^. After removing duplicate articles (n = 61), two independent and blinded investigators designated which studies should be considered for inclusion or exclusion by screening the titles and abstracts. Subsequently, the blinding of the Rayyan tool was removed to show the concordances and disagreements between the reviewers.

Cohen’s kappa index assessed inter-rater agreement ^46^ at a value of 0.845, indicating excellent agreement. A total of 33 articles met eligibility criteria and were read in full, except for one that was not retrieved. After full-text review, eight studies were excluded following discussion between the two researchers, the reasons of which are provided in Supplementary Material (Box S2; https://cadernos.ensp.fiocruz.br/static//arquivo/suppl-e00181525_1729.pdf). Any discrepancies were resolved by a third reviewer.

Studies identified by unsystematic searches and references from selected studies (n = 4) were also included to ensure a more comprehensive coverage, resulting in a final meta-synthesis corpus of 28 articles. Box 1 details information on the articles retrieved.

**Figure 1:**
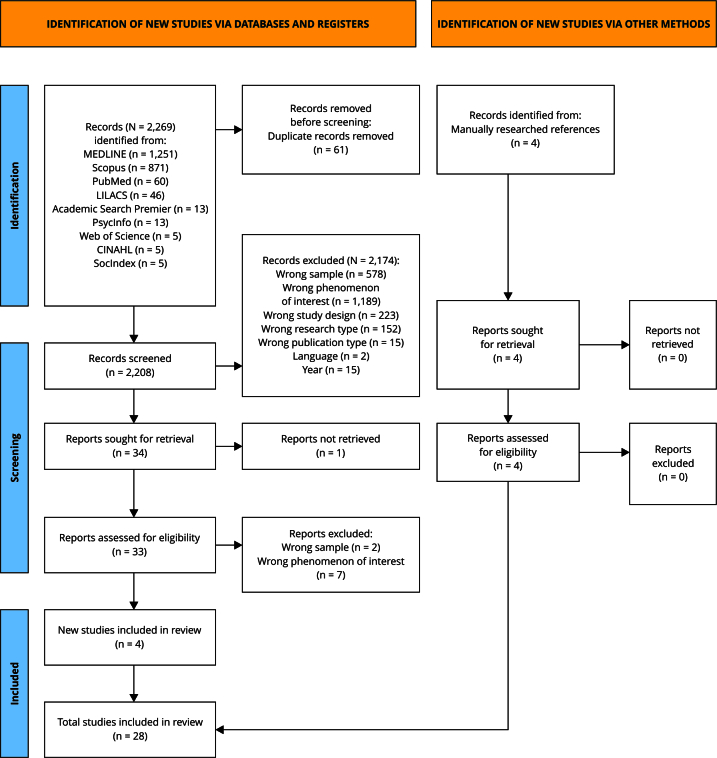
*Preferred Reporting Items for Systematic Reviews and Meta-Analyses* (PRISMA) diagram of study selection process.

Figure 1 brings a flowchart explaining the selection process and reasons for exclusion developed based on the *Preferred Reporting Items for Systematic Reviews and Meta-Analyses* (PRISMA) guidelines ^47^.

**Box 1:** Characteristics of the included studies (n = 28).

STUDY (YEAR)	AUTHORS’ COUNTRY OF ORIGIN	OBJECTIVE/RESEARCH QUESTION	THEORY	DATA COLLECTION	DATA ANALYSIS	FAMILY CHARACTERIZATION
Tuval-Mashiach et al. ^49^ (2013)	Israel	To explore how mothers perceive the impact of their eating disorder on motherhood and relationships with their children and families	ND	In-depth groups	Thematic analysis	Participants: 13 mothers; Age (years): 23-48; Diagnosis: bulimia nervosa (3), anorexia nervosa (4) and eating disorder not otherwise specified (6)
Svensson et al. ^50^ (2013)	Sweden	To investigate parental experiences of having and caring for a child with eating disorder	Phenomenological-hermeneutical	Semistructured interviews	Phenomenological-hermeneutical	Participants: 6 mothers and 4 fathers; Age (years): ND; Diagnosis: anorexia nervosa (4) and eating disorder not otherwise specified (3)
Bezance & Holliday ^51^ (2014)	United Kingdom	To explore the experiences of mothers receiving home treatment as part of treatment for their daughters	Phenomenological-hermeneutical	Semistructured interviews	Interpretative phenomenological analysis	Participants: 9 mothers; Age (years): 40-63; Diagnosis: anorexia nervosa
Patel et al. ^52^ (2014)	United States	To develop health communication messages for anorexia nervosa intervention to encourage parents to engage in self-care	Transactional model of stress and coping and the transtheoretical model of behavior change	Focus groups	Constant comparative method	Participants: 15 mothers and 4 fathers; Age (years): ND; Diagnosis: eating disorder
Stitt & Reupert ^53^ (2014)	Australia	To identify parents’ perceptions regarding the impact of eating disorder on their children and parenting	Phenomenological-hermeneutical	In-depth semistructured interviews	Interpretative phenomenological analysis	Participants: 9 mothers; Age (years): 20-49; Diagnosis: anorexia nervosa (4), bulimia nervosa (4) and eating disorder not otherwise specified (1)
Thomson et al. ^54^ (2014)	United Kingdom	To understand reasons for slow parental recognition that their child had anorexia nervosa eating problem and the process of deciding to seek help	Phenomenological-hermeneutical	Semistructured interviews	Interpretative phenomenological analysis	Participants: 7 mothers and 1 father; Age (years): 30-59; Diagnosis: anorexia nervosa
Leonidas & Santos ^55^ (2015)	Brazil	To investigate the social support network of women with eating disorders, emphasizing their affective family relations	Psychodynamics and transgenerational transmission	Semistructured interview, network’s map and genogram	Thematic analysis	Participants: 12 women with eating disorder; Age (years): 20-40; Diagnosis: anorexia nervosa (8) and bulimia nervosa (4)
Leonidas & Santos ^56^ (2015)	Brazil	To evaluate the transactional patterns in families of women with eating disorder using genogram	Systemic family therapy	Semistructured interview and genogram	Thematic analysis	Participants: 12 women with eating disorder; Age (years): 20-40; Diagnosis: anorexia nervosa (8) and bulimia nervosa (4)
McCormack & McCann ^57^ (2015)	Ireland	To investigate the subjective experiences of parents in caring for anorexia nervosa adolescent diagnosed with anorexia nervosa	ND	Semistructured interviews	Thematic analysis	Participants: 7 mothers and 3 fathers; Age (years): ND; Diagnosis: anorexia nervosa
Sadeh-Sharvit et al. ^58^ (2015)	Israel	To explore maternal feeding-related perceptions in mothers with eating disorder with toddler age children	Phenomenological-hermeneutical	Semistructured interviews	Interpretative phenomenological analysis	Participants: 29 mothers; Age (years): 31 (average); Diagnosis: anorexia nervosa (14), bulimia nervosa (13) and eating disorder not otherwise specified (2)
Fox & Whittlesea ^59^ (2017)	United Kingdom	To develop a grounded theory to explore caregivers’ responses to managing anorexia nervosa, focusing particularly on carers’ experience of accommodation	Grounded theory	Semistructured interviews	Grounded theory	Participants: 5 mothers, 2 fathers and 1 sister; Age (years): 49-74; Diagnosis: anorexia nervosa
Smalley et al. ^60^ (2017)	United Kingdom	To explore how women with anorexia nervosa perceive their family relationships, particularly focusing on triadic interactions, including the relationship between parents and their role in relation to the marital relationship	Attachment and family systems theory	Semistructured individual interviews, a family sculpt and *Adolescent Separation Anxiety Interview* (ASAI)	Interpretative phenomenological analysis and attachment analysis (modified Resnick analysis)	Participants: 6 women with eating disorder; Age (years): 16-18; Diagnosis: anorexia nervosa
Claydon et al. ^61^ (2018)	United States	To understand the intersection between eating disorder and pregnancy from the lived experience of women who have been pregnant or want to or do not want to become pregnant	Feminist theory	Interview and document analysis of diaries or blogs	Thematic analysis	Participants: 15 women with eating disorder; Age (years): 25-55; Diagnosis: anorexia nervosa, bulimia nervosa, eating disorder not otherwise specified and binge eating disorder
Fjermestad et al. ^62^ (2020)	Norway	To examine the experiences of siblings of patients with particularly severe anorexia nervosa, to further inform practitioners on how to tailor family-based treatment for anorexia nervosa	ND	Semistructured interviews	Systematic text condensation	Participants: 13 siblings; Age (years): 12-23; Diagnosis: anorexia nervosa
Williams et al. ^63^ (2020)	Canada	To investigate the experience of parents who discover their child was living with anorexia nervosa	Hermeneutic inquiry	Unstructured interviews	Hermeneutic inquiry	Participants: 9 mothers and 3 fathers; Age (years): 44-61; Diagnosis: anorexia nervosa
Cribben et al. ^64^ (2021)	United Kingdom	To develop a greater understanding of parents’ experiential perspective of eating disorder treatment in the United Kingdom, compared with guidances published by Beat and Academy for Eating Disorders	ND	Focus groups	Thematic analysis	Participants: 24 mothers and 8 fathers; Age (years): ND; Diagnosis: anorexia nervosa
Karlstad et al. ^65^ (2021)	Norway	To explore the experiences of parents with adult daughters suffering from anorexia nervosa and bulimia nervosa	Constructivist grounded theory	Semistructured interviews	Constructivist grounded theory	Participants: 7 mothers and 4 fathers; Age (years): 52-70; Diagnosis: anorexia nervosa (6) and bulimia nervosa (1)
Karlstad et al. ^66^ (2021)	Norway	To expand knowledge about the experiences and coping strategies of siblings of adult women with anorexia nervosa and bulimia nervosa	Constructivist grounded theory	Semistructured interviews	Constructivist grounded theory	Participants: 7 sisters and 3 brothers; Age (years): 20-31; Diagnosis: anorexia nervosa (6) and bulimia nervosa (2)
Kinnaird et al. ^67^ (2021)	United Kingdom	To explore the support needs of carers of loved ones with cooccuring autism spectrum condition and anorexia nervosa, and to investigate in more detail carer views on how this population can best be supported	ND	Semistructured peer interviews	Thematic analysis	Participants: 8 sisters and 3 brothers; Age (years): 38-74; Diagnosis: anorexia nervosa (10) and binge eating disorder (1)
Persico et al. ^68^ (2021)	France	To explore siblings’ representations of anorexia nervosa and to assess their emotional experiences in the support group named “sibling group” to improve group therapy system	Phenomenological approach	Focus groups	Interpretative phenomenological analysis	Participants: 4 sisters and 3 brothers; Age (years): 7-19; Diagnosis: anorexia nervosa
Valdanha-Ornelas et al. ^69^ (2021)	Brazil	To analyze the experiences of family relations from the perspective of a young woman diagnosed with bulimia nervosa, her mother and her father	Psychoanalytic approach	Semistructured interviews	Thematic analysis	Participants: 1 mother, 1 daughter with eating disorder and 1 father; Age (years): 24-50; Diagnosis: bulimia nervosa
Batchelor et al. ^70^ (2022)	United Kingdom	To explore the experiential perspectives of siblings and partners of a loved one with anorexia nervosa eating disorder, and compare this with the best-practice standards and healthcare rights published by Beat and Academy for Eating Disorders	ND	Focus groups	Thematic analysis	Participants: 9 sisters and 1 brother; Age (years): 24-50; Diagnosis: anorexia nervosa (13) and bulimia nervosa (2)
Karlstad et al. ^71^ (2022)	Norway	To generate a theory about how family life is afected by eating disorder	Constructivist grounded theory	Semistructured interviews	Constructivist grounded theory	Participants: 7 mother, 4 fathers, 7 sisters and 3 brothers; Age (years): 20-70; Diagnosis: anorexia nervosa (8) and bulimia nervosa (3)
Konstantellou et al. ^72^ (2022)	United Kingdom	To explore how parents of young people with a restrictive eating disorder experience and manage uncertainty	Phenomenological approach	Focus groups	Interpretative phenomenological analysis	Participants: 9 mothers and 8 fathers; Age (years): ND; Diagnosis: anorexia nervosa (10) and eating disorder not otherwise specified (3)
Saunokonoko et al. ^73^ (2022)	Australia	To explore the father-daughter relationship where bulimia nervosa has arisen in the daughter	Hermeneutic phenomenological	Unstructured interviews	Phenomenological-hermeneutical	Participants: 3 women with bulimia nervosa; Age (years): ND; Diagnosis: bulimia nervosa
Simões & Santos ^74^ (2022)	Brazil	To comprehend the father-child bond and the paternity of a father of a young adult man in treatment for bulimia nervosa at a specialized service	Psychoanalysis of linking configurations	Genogram and semistructured interview	Thematic analysis	Participants: 1 father; Age (years): 61; Diagnosis: bulimia nervosa
Elwyn et al. ^75^ (2024)	Australia	To explore the experiences of two twin pairs discordant for anorexia nervosa and explores factors infuencing the development and persistence of anorexia nervosa	Phenomenological approach	Collaborative autoethnography and unstructured online meetings	Thematic analysis	Participants: 4 brothers; Age (years): 26-33; Diagnosis: anorexia nervosa
O’Sullivan et al. ^76^ (2024)	Ireland	To explore the perspectives of fathers regarding family involvement in the treatment of children and young people diagnosed with eating disorder	ND	Focus groups	Qualitative content analysis	Participants: 7 fathers; Age (years): ND; Diagnosis: eating disorder

ND: not described.

### Methodological quality appraisal of the articles

The methodological quality of the studies was assessed using the *CASP Qualitative Checklist* ^41^, which evaluates key aspects of qualitative research, including: objective and its relevance, methodology, the clarity of ethical procedures, and data presentation. Two independent researchers conducted the assessments and resolved discrepancies by consensus. Methodological consistency of all studies was considered appropriate for qualitative research, showing consistency across almost all findings, except for ethical considerations, in which inconsistencies were identified in 12 studies. Box 2 presents this process in detail.

**Box 2:** Quality appraisal of included studies according to the *Critical Appraisal Skills Program* (CASP).

STUDY (YEAR)	QUESTIONS									
	1	2	3	4	5	6	7	8	9	10
Tuval-Mashiach et al. ^49^ (2013)	Yes	Yes	Yes	Yes	Yes	No	Yes	Yes	Yes	Yes
Svensson et al. ^50^ (2013)	Yes	Yes	Yes	Yes	Yes	No	Yes	Yes	Yes	Yes
Bezance & Holliday ^51^ (2014)	Yes	Yes	Yes	Yes	Yes	Yes	Yes	Yes	Yes	Yes
Patel et al. ^52^ (2014)	Yes	Yes	Yes	Yes	Yes	Can’t tell	Yes	Yes	Yes	Yes
Stitt & Reupert ^53^ (2014)	Yes	Yes	Yes	Yes	Yes	Yes	Yes	Yes	Yes	Yes
Thomson et al. ^54^ (2014)	Yes	Yes	Yes	Yes	Yes	Yes	Yes	Yes	Yes	Yes
Leonidas & Santos ^55^ (2015)	Yes	Yes	Yes	Yes	Yes	No	Yes	Yes	Yes	Yes
Leonidas & Santos ^56^ (2015)	Yes	Yes	Yes	Yes	Yes	No	Yes	Yes	Yes	Yes
McCormack & McCann ^57^ (2015)	Yes	Yes	Yes	Yes	Yes	No	Yes	Yes	Yes	Yes
Sadeh-Sharvit et al. ^58^ (2015)	Yes	Yes	Yes	Yes	Yes	No	Yes	Yes	Yes	Yes
Fox & Whittlesea ^59^ (2017)	Yes	Yes	Yes	Yes	Yes	Yes	Yes	Yes	Yes	Yes
Smalley et al. ^60^ (2017)	Yes	Yes	Yes	Yes	Yes	Yes	Yes	Yes	Yes	Yes
Claydon et al. ^61^ (2018)	Yes	Yes	Yes	Yes	Yes	Yes	Yes	Yes	Yes	Yes
Fjermestad et al. ^62^ (2020)	Yes	Yes	Yes	Yes	Yes	Can’t tell	Yes	Yes	Yes	Yes
Williams et al. ^63^ (2020)	Yes	Yes	Yes	Yes	Yes	Yes	Yes	Yes	Yes	Yes
Cribben et al. ^64^ (2021)	Yes	Yes	Yes	Yes	Yes	Yes	Yes	Yes	Yes	Yes
Karlstad et al. ^65^ (2021)	Yes	Yes	Yes	Yes	Yes	Yes	Yes	Yes	Yes	Yes
Karlstad et al. ^66^ (2021)	Yes	Yes	Yes	Yes	Yes	Yes	Yes	Yes	Yes	Yes
Kinnaird et al. ^67^ (2021)	Yes	Yes	Yes	Yes	Yes	Yes	Yes	Yes	Yes	Yes
Persico et al. ^68^ (2021)	Yes	Yes	Yes	Yes	Yes	No	Yes	Yes	Yes	Yes
Valdanha-Ornelas et al. ^69^ (2021)	Yes	Yes	Yes	Yes	Yes	No	Yes	Yes	Yes	Yes
Batchelor et al. ^70^ (2022)	Yes	Yes	Yes	Yes	Yes	Yes	Yes	Yes	Yes	Yes
Karlstad et al. ^71^ (2022)	Yes	Yes	Yes	Yes	Yes	Yes	Yes	Yes	Yes	Yes
Konstantellou et al. ^72^ (2022)	Yes	Yes	Yes	Yes	Yes	Yes	Yes	Yes	Yes	Yes
Saunokonoko et al. ^73^ (2022)	Yes	Yes	Yes	Yes	Yes	Yes	Yes	Yes	Yes	Yes
Simões & Santos ^74^ (2022)	Yes	Yes	Yes	Yes	Yes	No	Yes	Yes	Yes	Yes
Elwyn et al. ^75^ (2024)	Yes	Yes	Yes	No	Yes	Yes	Yes	Yes	Yes	Yes
O’Sullivan et al. ^76^ (2024)	Yes	Yes	Yes	Yes	Yes	Can’t tell	Yes	Yes	Yes	Yes

Questions: (1) Was there a clear statement of the aims of the research?; (2) Is a qualitative methodology appropriate?; (3) Was the research design appropriate to address the aims of the research?; (4) Was the recruitment strategy appropriate to the aims of the research?; (5) Were the data collected in a way that addressed the research issue?; (6) Has the relationship between researcher and participants been adequately considered?; (7) Have ethical issues been taken into consideration?; (8) Was the data analysis sufficiently rigorous?; (9) Is there a clear statement of findings?; (10) How valuable is the research?

### Data analysis

Data were analyzed using the reflexive thematic analysis method according to the following structure ^35^: (1) Full reading of the texts performed using QDA Miner Lite software to generate codes through line-by-line analysis; (2) Reviewing and grouping the codes into similar themes, which formed the descriptive categories derived from the original data of primary studies; (3) Developing analytical themes through interpretation of these categories. The process was discussed and validated by the research team.

Confidence in the review findings was assessed by GRADE-CERQual (https://www.cerqual.org/) ^48^. This approach focuses on four key components: methodological limitations, coherence, data adequacy, and relevance of the findings from the included studies to the review question. Confidence in each finding can be rated at one of four levels: very low, low, moderate, or high.

## Results

Most of the 28 articles ^49^ ^,^ ^50^ ^,^ ^51^ ^,^ ^52^ ^,^ ^53^ ^,^ ^54^ ^,^ ^55^ ^,^ ^56^ ^,^ ^57^ ^,^ ^58^ ^,^ ^59^ ^,^ ^60^ ^,^ ^61^ ^,^ ^62^ ^,^ ^63^ ^,^ ^64^ ^,^ ^65^ ^,^ ^66^ ^,^ ^67^ ^,^ ^68^ ^,^ ^69^ ^,^ ^70^ ^,^ ^71^ ^,^ ^72^ ^,^ ^73^ ^,^ ^74^ ^,^ ^75^ ^,^ ^76^ were conducted in the United Kingdom (n = 8) ^51^ ^,^ ^54^ ^,^ ^59^ ^,^ ^60^ ^,^ ^64^ ^,^ ^67^ ^,^ ^70^ ^,^ ^72^, followed by Brazil (n = 4) ^55^ ^,^ ^56^ ^,^ ^69^ ^,^ ^74^ and Norway (n = 4) ^62^ ^,^ ^65^ ^,^ ^66^ ^,^ ^71^. Other countries included Australia (n = 3) ^53^ ^,^ ^73^ ^,^ ^75^, Israel (n = 2) ^49^ ^,^ ^58^, the United States (n = 2) ^52^ ^,^ ^61^, Ireland (n = 2) ^57^ ^,^ ^76^, Sweden (n = 1) ^50^, Canada (n = 1) ^63^, and France (n = 1) ^68^.

Most studies used individual interviews (n = 21) ^50^ ^,^ ^51^ ^,^ ^53^ ^,^ ^54^ ^,^ ^55^ ^,^ ^56^ ^,^ ^57^ ^,^ ^58^ ^,^ ^59^ ^,^ ^60^ ^,^ ^61^ ^,^ ^62^ ^,^ ^63^ ^,^ ^65^ ^,^ ^66^ ^,^ ^67^ ^,^ ^69^ ^,^ ^71^ ^,^ ^73^ ^,^ ^74^ ^,^ ^75^ for data collection, whereas only seven employed focus group ^49^ ^,^ ^52^ ^,^ ^64^ ^,^ ^68^ ^,^ ^70^ ^,^ ^72^ ^,^ ^76^. Notable among data analysis approached were thematic analysis (n = 11) ^49^ ^,^ ^55^ ^,^ ^56^ ^,^ ^57^ ^,^ ^61^ ^,^ ^64^ ^,^ ^67^ ^,^ ^69^ ^,^ ^70^ ^,^ ^74^ ^,^ ^75^ and interpretative phenomenological analysis (n = 7) ^51^ ^,^ ^53^ ^,^ ^54^ ^,^ ^58^ ^,^ ^60^ ^,^ ^68^ ^,^ ^72^. The review sample comprised 322 participants - 157 mothers, 50 fathers, 66 siblings, and 49 women diagnosed with eating disorders.

Analysis of the 28 studies generated 110 codes during the initial open coding phase. After review, the remaining 47 codes were organized into four descriptive themes: (1) Anorexia nervosa/bulimia nervosa within the family environment: meanings attributed to the symptoms; (2) Anorexia nervosa/bulimia nervosa dominance within the family system: care that also causes harm; (3) Challenges in accessing care: individual and social barriers; (4) Pathways to coping: supporting the whole family - a public health issue. From this, two analytical themes were developed: (1) Interplay between anorexia/bulimia nervosa and family relations: a complex relationship that feeds on itself; (2) A comprehensive approach to care that embraces all family members. Box 3 details the results.

Overall, quality assessment of the included studies provided moderate confidence for the review findings. However, many studies poorly discussed or failed to address the reflexivity of the research process. Differences in sample sizes were observed regarding gender distribution, with men (fathers or brothers) being underrepresented compared with women. Box 4 provides the GRADE-CERQual assessment for each finding.

**Box 3:** Codes and themes generated by the thematic synthesis process.

CODES (LINE-BY-LINE CODING)	DESCRIPTIVE THEMES	ANALYTICAL THEMES
Fragile and conflicted family dynamics	(1) Anorexia nervosa/bulimia nervosa within the family environment: meanings attributed to the symptoms	(1) Interplay between anorexia/bulimia nervosa and the family relations: a complex relationship that feeds on itself (2) A comprehensive approach to care that embraces all family members
Mother-child enmeshment		
Distancing from the father figure		
Vulnerability of the marital bond		
Family inheritance		
Symptom as a defensive mechanism		
Eating disorder as identify		
Trapped in their own suffering	(2) Anorexia nervosa/bulimia nervosa dominance within the family system: care that also causes harm	
The one who turned into a stranger		
A weakened bond with the ill person		
Turbulent and chaotic family environment		
Hostage family		
Financial and professional impacts		
Weakening of communication		
Fragmentation of the family		
Loneliness that affects everyone		
Concern and fear		
The conflicting emotions of caregiving		
Unconditional care		
To shoulder a responsibility that isn’t yours		
The burden of caregiving		
The powerlessness in caregiving		
Guilt over failing in their caregiving role		
The forgotten sibling		
Social pressure	(3) Challenges in accessing care: individual and social barriers	
Difficulty in recognition		
Lack of knowledge		
The secret of the unspoken		
Denial of the disease		
Treatment resistance		
Lack of adequate professional support		
Better understanding and acceptance	(4) Pathways to coping: supporting the whole family − a public health issue	
Improving communication		
Different ways of caring		
Self-care		
Distancing as a form of self-protection		
Hope		
Personal autonomy in help-seeking		
Emotional development of family members		
Motherhood as a motivation for treatment		
Strengthening of family bonds		
Support from the social network		
Support from health services		

**Box 4:** CERQual evidence profile.

SUMMARY OF REVIEW FINDING	STUDIES CONTRIBUTING TO THE REVIEW FINDING	METHODOLOGICAL LIMITATIONS *	COHERENCE **	ADEQUACY ***	RELEVANCE ^#^	CERQual ASSESSMENT OF CONFIDENCE IN THE EVIDENCE	EXPLANATION OF CERQual ASSESSMENT
Descriptive theme 1 − Anorexia nervosa/bulimia nervosa within the family environment: meanings attributed to the symptoms							
Families affected by eating disorder are often characterized by fragile or conflicted emotional bonds. The maternal bond is marked by symbiotic dynamic, while the paternal relationship is frequently described as distant and absent, and marked by significant conflict	^49^ ^,^ ^51^ ^,^ ^55^ ^,^ ^56^ ^,^ ^60^ ^,^ ^69^ ^,^ ^73^ ^,^ ^74^ ^,^ ^76^	No or very minor concerns regarding methodological limitations. Five ^49^ ^,^ ^55^ ^,^ ^56^ ^,^ ^74^ ^,^ ^76^ of the 9 studies did not consider the relationship between researcher and participants	Minor concerns were noted in cases where the mother-child bond was described as indifferent rather than symbiotic ^49^, and where parental bonds were generally reported as positive ^55^ ^,^ ^56^	Minor concerns regarding adequacy. Two studies that contributed to the finding on the paternal relationship from the perspective of fathers had 1 ^74^ and 7 ^76^ fathers as participants. Although the data presented by the studies is rich, this may suggest a lack of studies focused primarily on the father’s perspectives	Minor concerns due to the absence of sibling participants. Findings was observed across a variety of countries, contexts, and age groups	Moderate confidence: it is likely that the review finding provides an accurate representation of the phenomenon of interest	There are some minor concerns regarding a few disconfirming cases, limited information on the perspectives of fathers, and no information on siblings
A weakened marital bond, with the child either mediating parental conflicts or having their illness used to deflect the parents from addressing their own marital issues	^55^ ^,^ ^60^ ^,^ ^69^	No or very minor concerns regarding methodological limitations. Two ^55^ ^,^ ^69^ of the 3 studies did not consider the relationship between researcher and participants	Minor concerns, given that in some cases the daughter either avoided intervening in or did not recognize parental conflict, depending on her attachment strategies − particularly among those with dismissing attachment styles ^60^	No or very minor concerns regarding adequacy. One ^69^ of the 3 studies had 3 participants. However, the data is rich and the study’s design was well aligned with this particular finding	There are minor concerns that this finding may be specific to women with eating disorder and was observed only in Brazil ^55^ ^,^ ^69^ and the United Kingdom ^60^	Moderate confidence: it is likely that the review finding provides an accurate representation of the phenomenon of interest	There are minor concerns due to a few disconfirming cases, the possibility that this finding is specific to mothers, and the fact that it was observed in only a few countries
Parents’ dysfunctional attitudes and behaviors related to food and body image	^57^ ^,^ ^61^ ^,^ ^69^ ^,^ ^73^ ^,^ ^74^ ^,^ ^75^	Moderate concerns regarding methodological limitations. Two ^57^ ^,^ ^69^ of the 6 studies did not consider the relationship between researcher and participants. One study ^74^, in which the participants were authors themselves, did not provide sufficient information nor discussions on its particular recruitment strategy	Minor concerns because the influence of this finding is unclear in some examples, as it does not specify the extent to which parental behavior affects the daughter’s symptoms ^69^	Moderate concerns regarding adequacy. Three ^69^ ^,^ ^73^ ^,^ ^75^ of the 6 studies had insufficient sample sizes. Although the studies’ design and collected data are rich in terms of explaining the phenomenon of interest, larger sample sizes could provide better insight on this particular topic	No or very minor concerns. This finding was identified across diverse countries, contexts, family members, and age groups	Low confidence: it is possible that the review finding provides an accurate representation of the phenomenon of interest	There are some concerns regarding methodological limitations and small sample sizes, with minor concerns about the unclear influence of the finding on the phenomenon
Use of the symptom as a way to avoid conflict and cope with trauma − providing a sense of control, familiarity, and emotional comfort − as well as a means to preserve identity	^55^ ^,^ ^60^ ^,^ ^72^ ^,^ ^73^ ^,^ ^75^	No or very minor concerns regarding methodological limitations. One study ^75^, in which the participants were authors themselves, did not provide sufficient information nor discussions on its particular recruitment strategy	No or very minor concerns, as it is considered to be well grounded in the data from the contributing studies	No or very minor concerns regarding adequacy	No or very minor concerns. This finding was identified across diverse countries, contexts, family members, and age groups	High confidence: it is highly likely that the review finding provides an accurate representation of the phenomenon of interest	Despite minor methodological limitations in one study, this finding was consistently identified across diverse contexts
Descriptive theme 2 − Anorexia nervosa/bulimia nervosa dominance within the family system: care that also causes harm							
The intense emotional suffering caused by the eating disorder consumes and constrains the person’s entire life, affecting their emotional bonds, including the experience of motherhood	^49^ ^,^ ^53^ ^,^ ^55^ ^,^ ^56^ ^,^ ^57^ ^,^ ^61^ ^,^ ^68^ ^,^ ^75^	Minor concerns regarding methodological limitations. Five ^49^ ^,^ ^55^ ^,^ ^56^ ^,^ ^58^ ^,^ ^68^ of the 8 studies did not consider the relationship between researcher and participants. One study _^(74)^_ , in which the participants were authors themselves, did not provide sufficient information nor discussions on its particular recruitment strategy	No or very minor concerns as it is considered to be well grounded in the data from the contributing studies	No or very minor concerts regarding adequacy. Although 1 ^75^ study’s data raised concerns regarding sample size and design adequacy, the collected data was sufficient and aligned with the other studies that contributed to this finding	No or very minor concerns due to the absence of father participants. However, the finding was observed across a variety of countries, contexts, and age groups	High confidence: it is highly likely that the review finding provides an accurate representation of the phenomenon of interest	Despite minor methodological limitations, this finding was consistently identified across diverse contexts
The emotional impacts on all family members include a sense of unfamiliarity and distrust toward the affected individual; a turbulent, conflict-ridden home environment with impaired communication; feelings of loneliness and worry; and the substantial caregiving burden, which leads to emotional exhaustion, powerlessness, and the neglect of other family members	^49^ ^,^ ^50^ ^,^ ^51^ ^,^ ^52^ ^,^ ^53^ ^,^ ^54^ ^,^ ^55^ ^,^ ^57^ ^,^ ^59^ ^,^ ^60^ ^,^ ^62^ ^,^ ^63^ ^,^ ^64^ ^,^ ^65^ ^,^ ^66^ ^,^ ^67^ ^,^ ^68^ ^,^ ^69^ ^,^ ^70^ ^,^ ^71^ ^,^ ^72^ ^,^ ^75^ ^,^ ^76^	No or very minor concerns regarding methodological limitations. Nine ^49^ ^,^ ^50^ ^,^ ^52^ ^,^ ^55^ ^,^ ^57^ ^,^ ^62^ ^,^ ^68^ ^,^ ^69^ ^,^ ^76^ of the 23 studies did not consider the relationship between researcher and participants. One study _^(75)^_ , in which the participants were authors themselves, did not provide sufficient information nor discussions on its particular recruitment strategy	No or very minor concerns, as it is considered to be well grounded in the data from the contributing studies	Minor concerns regarding adequacy. Although the overall data is adequate and sufficient in terms of sample sizes and data richness, it should be noted that men’s perspective on this phenomenon was commonly underrepresented through smaller sample sizes when compared to women’s in 17 studies ^49^ ^,^ ^50^ ^,^ ^51^ ^,^ ^52^ ^,^ ^53^ ^,^ ^54^ ^,^ ^55^ ^,^ ^57^ ^,^ ^59^ ^,^ ^60^ ^,^ ^62^ ^,^ ^63^ ^,^ ^65^ ^,^ ^66^ ^,^ ^67^ ^,^ ^68^ ^,^ ^71^. It should also be noted, however, that one study _^(75)^_ focused specifically on fatherhood and provided data that fostered valuable contributions towards the construction of this finding	No or very minor concerns. This finding was identified across diverse countries, contexts, family members, and age groups	High confidence: it is highly likely that the review finding provides an accurate representation of the phenomenon of interest	Although there were minor concerns regarding data adequacy in some studies, related to the underrepresentation of the male population, the findings were consistently identified across diverse contexts
Descriptive theme 3 − Challenges in accessing care: individual and social barriers							
The challenges faced by families in seeking care included societal pressure from beauty standards that idealize thinness; difficulty recognizing the disorder’s severity; stigma and limited public understanding; the burden of secrecy; treatment resistance; and inadequate healthcare services	^49^ ^,^ ^50^ ^,^ ^51^ ^,^ ^52^ ^,^ ^53^ ^,^ ^54^ ^,^ ^55^ ^,^ ^56^ ^,^ ^57^ ^,^ ^58^ ^,^ ^59^ ^,^ ^60^ ^,^ ^61^ ^,^ ^62^ ^,^ ^63^ ^,^ ^64^ ^,^ ^65^ ^,^ ^66^ ^,^ ^67^ ^,^ ^68^ ^,^ ^69^ ^,^ ^70^ ^,^ ^71^ ^,^ ^72^ ^,^ ^75^	Moderate concerns regarding methodological limitations. Eight ^49^ ^,^ ^50^ ^,^ ^52^ ^,^ ^55^ ^,^ ^56^ ^,^ ^62^ ^,^ ^68^ ^,^ ^69^ of the 25 studies did not consider the relationship between researcher and participants. Considering the particularity of this review finding, the lack of discussions and reflexivity about the relationship between researchers in these 8 studies may have compromised the collection, analysis and quality of data. It also raises ethical concerns regarding participant’s wellness during and after the research was conducted. One study ^75^, in which the participants were authors themselves, did not provide sufficient information nor discussions on its particular recruitment strategy	Moderate concerns due to some disconfirming cases in which healthcare services positively influence treatment ^50^ ^,^ ^51^ ^,^ ^57^ ^,^ ^61^ ^,^ ^62^ ^,^ ^64^ ^,^ ^68^ ^,^ ^70^ ^,^ ^75^	No or very minor concerts regarding adequacy	No or very minor concerns. This finding was identified across diverse countries, contexts, family members, and age groups	Low confidence: it is possible that the review finding provides an accurate representation of the phenomenon of interest	There are some concerns regarding methodological limitations and a few disconfirming cases related to the performance of healthcare teams and services, which require careful analysis to assess their influence on the phenomenon
Descriptive theme 4 − Pathways to coping: supporting the whole family − a public health issue							
Families used various strategies to cope with eating disorder, including accepting the diagnosis; fostering open communication; implementing care approaches beyond food-related issues; prioritizing self-care and routines; hope for recovery and autonomy; and strengthening family bonds; support networks; and engagement with healthcare services	^49^ ^,^ ^50^ ^,^ ^51^ ^,^ ^52^ ^,^ ^53^ ^,^ ^54^ ^,^ ^55^ ^,^ ^56^ ^,^ ^57^ ^,^ ^58^ ^,^ ^60^ ^,^ ^61^ ^,^ ^62^ ^,^ ^63^ ^,^ ^64^ ^,^ ^65^ ^,^ ^66^ ^,^ ^67^ ^,^ ^68^ ^,^ ^69^ ^,^ ^70^ ^,^ ^71^ ^,^ ^72^ ^,^ ^73^ ^,^ ^74^ ^,^ ^75^ ^,^ ^76^	Moderate concerns regarding methodological limitations. Eleven ^49^ ^,^ ^50^ ^,^ ^52^ ^,^ ^55^ ^,^ ^56^ ^,^ ^58^ ^,^ ^62^ ^,^ ^68^ ^,^ ^69^ ^,^ ^74^ ^,^ ^76^ of 27 studies did not consider the relationship between researcher and participants. One of the dimensions of this finding deals with seeking care through relationships with family members and healthcare services, the lack of reflexivity may have compromised data collection and analysis on this particular theme	No or very minor concerns, as it is considered to be well grounded in the data from the contributing studies	No or very minor concerns regarding adequacy. Although their data was rich and aligned with this findings’ theme, 4 studies ^69^ ^,^ ^73^ ^,^ ^74^ ^,^ ^75^ had sample sizes lower than 4	No or very minor concerns. This finding was identified across diverse countries, contexts, family members, and age groups	Moderate confidence: it is likely that the review finding provides an accurate representation of the phenomenon of interest	Despite minor methodological limitations, this finding was consistently identified across diverse contexts

Source: prepared by authors.

* Methodological limitations: concerns about the design or conduct of included studies;

** Coherence: the degree to which the findings are well supported by the data and show a clear and consistent pattern across studies;

*** Data adequacy: the richness and quantity of data supporting the finding;

^#^ Relevance of the findings from the included studies to the review question.

### Descriptive theme 1 - Anorexia nervosa/bulimia nervosa within the family environment: meanings attributed to the symptoms

This theme explores the interplay between family dynamics and anorexia nervosa/bulimia nervosa, analyzing how they influence one other in a complex manner without a clear distinction between cause and effect. Studies indicate that families affected by eating disorders often present weak or conflicted emotional bonds which may precede the onset of clinical symptoms ^49^ ^,^ ^55^ ^,^ ^56^ ^,^ ^69^ ^,^ ^73^. These families display extreme patterns - from high demands to emotional neglect, leading to poor communication and low cohesion.

With anorexia nervosa/bulimia nervosa onset, the mother-daughter bond deepened significantly (“*But now we’re really, really, really close, and she’s the person I’m closest to in the world*” ^60^ [p. 36]), creating intense emotional interdependence ^49^ ^,^ ^51^ ^,^ ^55^ ^,^ ^56^ ^,^ ^60^ ^,^ ^69^. However, in this symbiotic dynamic, the lack of clearly defined boundaries between mother and daughter creates a dependency so severe that any attempt at separation fuels in the mother as a profound sense of betrayal or disappointment, evoking ambivalent feelings in the daughter: “*...I feel kind of bitter toward my mother, you know. I can’t really explain what this feeling is. Maybe resentment, a mix of resentment and sadness for the things she has put me through*” ^69^ (p. 6, our translation).

In contrast, patients’ relationship with the paternal figure was often characterized as distant and absent, yet marked by significant conflict ^55^ ^,^ ^56^ ^,^ ^60^ ^,^ ^69^ ^,^ ^73^ ^,^ ^74^ ^,^ ^76^. Studies noted poor father-daughter compatibility since “*a man will never understand a woman’s insecurities*” ^60^ (p. 36). Fathers tended to adopt a pragmatic caregiving approach, causing frustration and tension: “*...*[I] *felt like a lot of anger towards him* [father] *and I don’t know whether that’s because he’s been telling me practically the things which I should be doing*” ^60^ (p. 37).

Living with an authoritarian and violent paternal figure profoundly impacted the lives of many participants, leading to traumatic events that can negatively affect their children’s emotional development as illustrated by one participant, who shared a traumatic experience involving a meal scene: “*I looked at him and I was like, ‘Dad, I don’t eat fish.’ And he was like, ‘Well, you’re going to eat it.’* (...) *And he shouted at me and made me eat it*” ^73^ (p. 8).

Deterioration of the marital bond also seems to be recurrent ^55^ ^,^ ^60^ ^,^ ^69^, with children often positioned at the center of parental conflicts, either mediating disputes or having their illness used to avoid the couple’s own issues, which “*makes it quite difficult for all of us to let go of the anorexia*” ^60^ (p. 38). This entanglement is often distressing, as one daughter put it: “*I literally step in as their therapist*” ^60^ (p. 38).

Moreover, studies suggest that the parents of individuals with eating disorder often have a background of dysfunctional attitudes and behaviors related to food and body image ^57^ ^,^ ^61^ ^,^ ^69^ ^,^ ^73^ ^,^ ^74^ ^,^ ^75^. These patterns reflect a family inheritance in which eating habits, body perception, and emotional expression are passed down across generations: “*I think part of the reason that I feel like I have a weight problem is because my mother had a weight problem*” ^61^ (p. 9).

Conversely, many participants acknowledged using their symptoms to avoid conflict and cope with trauma ^55^ ^,^ ^60^ ^,^ ^72^ ^,^ ^73^ ^,^ ^75^. One described anorexia nervosa as “*desirable*” ^60^ (p. 37) during arguments, offering control and familiarity. In this context, food also provided comfort and emotional security - “*to hold it and feel comforted that it was still there*” ^75^ (p. 12). Others saw eating disorder as a way to assert identity and resist expectations ^60^ ^,^ ^75^: “*...I was kind of just a good girl and I think now this is the first time that I’ve ever not done what I was told*” ^60^ (p. 37).

### Descriptive theme 2 - Anorexia nervosa/bulimia nervosa dominance within the family system: care that also causes harm

This topic examines how anorexia nervosa/bulimia nervosa gradually come to dominate family dynamics, with its chronicity reshaping bonds and caregiving roles. Individuals often feel trapped in ongoing suffering ^49^ ^,^ ^53^ ^,^ ^55^ ^,^ ^56^ ^,^ ^58^ ^,^ ^61^ ^,^ ^68^ ^,^ ^72^ ^,^ ^75^: “*as years passed with anorexia nervosa, I felt trapped and traumatized from being unable to heal and recover*” ^75^ (p. 10). A hard-to-break cycle given the emotional complexity sustaining it. One sister observed a perverse sense of pleasure in maintaining this pattern: “*...*[she] *feels she deserves it, and so the fact of inflicting that sort of thing on yourself, it gives you a good feeling, in the sense that you deserve it...*” ^68^ (p. 5).

When the disorder affects adult women, especially mothers, symptom manifestation often becomes apparent within their motherhood experiences. In these cases, the disordered eating can significantly undermine maternal caregiving ^49^ ^,^ ^53^ ^,^ ^55^ ^,^ ^56^ ^,^ ^58^ ^,^ ^61^. As one participant reflected: “*getting kind of obsessed with the idea of being perfect. Perfect lover. Perfect wife. Perfect mother. Perfect anorexic*” ^61^ (p. 8).

Family members often experienced the anorexia nervosa/bulimia nervosa onset with a profound sense of estrangement ^50^ ^,^ ^51^ ^,^ ^59^ ^,^ ^62^, as if “*she was a completely different person*” ^62^ (p. 84) or as “*the devil that’s got into my daughter*” ^51^ (p. 293). This produced increasingly fragile bonds, as emotional distance and frustration over denial or dishonesty about the illness weakened family connections ^59^ ^,^ ^62^ ^,^ ^64^ ^,^ ^66^ ^,^ ^69^.

As the eating disorder progressed, the home became chaotic ^49^ ^,^ ^50^ ^,^ ^54^ ^,^ ^57^ ^,^ ^59^ ^,^ ^62^ ^,^ ^66^ ^,^ ^69^ ^,^ ^70^ and “*horrendous*” ^57^ (p. 144). One sister shared: “*...suddenly it was okay to shout and scream at home, over every little thing. It is as though this one person* [the sister with the illness] *changed the rest of the family...*” ^66^ ^)^ (p. 4). Daily routines were dominated by eating disorder-related demands ^50^ ^,^ ^51^ ^,^ ^57^ ^,^ ^59^ ^,^ ^62^ ^,^ ^65^ ^,^ ^71^ ^,^ ^75^, with mealtimes becoming especially stressful: “*It affects everything, from morning to evening. Dinner, diets, meals...*” ^65^ (p. 5). Some families also faced serious financial and work disruptions due to treatment costs and caregiving responsabilities ^50^ ^,^ ^51^ ^,^ ^53^ ^,^ ^57^ ^,^ ^64^ ^,^ ^65^ ^,^ ^67^ ^,^ ^72^: “*It is like a second mortgage*” ^57^ (p. 144).

Researchers point out that family communication is also significantly affected ^57^ ^,^ ^62^ ^,^ ^66^ ^,^ ^68^ ^,^ ^69^ ^,^ ^76^, requiring cautious dialogue or avoidance of certain topics to prevent conflict and emotional distress: “*you’d have to think about what you are going to say before you say it*” ^76^ (p. 997). Over time, this can result in weakened relationships, creating distance and an “*empty space*” ^62^ (p. 86) among family members ^50^ ^,^ ^54^ ^,^ ^59^ ^,^ ^62^ ^,^ ^67^.

Many participants reported deep loneliness and isolation ^50^ ^,^ ^51^ ^,^ ^53^ ^,^ ^54^ ^,^ ^55^ ^,^ ^57^ ^,^ ^60^ ^,^ ^62^ ^,^ ^63^ ^,^ ^64^ ^,^ ^68^ ^,^ ^71^ ^,^ ^75^. One shared how the diagnosis created distance from her family: “*It’s like somebody put a box over you...*” ^60^ (p. 37). Another noted the ill person withdrawing into isolation: “*...you shut yourself up, inside your own bubble...*” ^68^ (p. 5). Families also avoid social interactions - no longer traveling, hosting friends, or going out to eat, “*because we wouldn’t leave her on her own*” ^51^ (p. 391).

Families often felt constant worry and fear about symptom severity ^50^ ^,^ ^51^ ^,^ ^57^ ^,^ ^62^ ^,^ ^63^ ^,^ ^64^ ^,^ ^68^ ^,^ ^69^ ^,^ ^72^ ^,^ ^75^. They feared both physical decline and uncertainty about recovery: “*there will always be signs... more on the inside with the after-effects. Because on the outside, they can be wiped out if you want...*” ^68^ (p. 5). Uncertainty about managing the emotional and relational challenges in caregiving increased this concern ^50^ ^,^ ^51^ ^,^ ^59^ ^,^ ^64^ ^,^ ^65^ ^,^ ^67^ ^,^ ^71^ ^,^ ^72^ ^,^ ^76^. Many struggled between accommodating the symptoms and confronting the disorder, as one participant expressed: “*...you want them to get better... But you also want peace...*” ^59^ (p. 494). This dilemma often caused significant strain: “*uncertainty can be imprisoning*” ^72^ (p. 1343).

In response to this fear, parents felt a deep urge to protect their children ^51^ ^,^ ^52^ ^,^ ^54^ ^,^ ^57^ ^,^ ^59^ ^,^ ^72^ ^,^ ^75^: “*I think it’s because it’s your child - you would do anything. It’s such a powerful instinct to protect them...*” ^72^ (p. 1343). But this resulted in caregiving beyond typical parental roles ^49^ ^,^ ^53^ ^,^ ^62^ ^,^ ^65^ ^,^ ^66^ ^,^ ^70^ ^,^ ^71^ ^,^ ^75^: “*I felt I had to take on so many responsibilities that were far beyond being a mum*” ^65^ (p. 5). At times, this imbalance sometimes led to role reversal, with children caring for their parents. As one mother shared, her child would say: “*C’mon, up you get, let’s go and have a shower...*” ^53^ (p. 513).

Caregivers often became overwhelmed ^50^ ^,^ ^51^ ^,^ ^52^ ^,^ ^59^ ^,^ ^62^ ^,^ ^63^ ^,^ ^64^ ^,^ ^65^ ^,^ ^67^ ^,^ ^68^ ^,^ ^69^ ^,^ ^70^ ^,^ ^71^ ^,^ ^72^ ^,^ ^75^ ^,^ ^76^, describing emotional exhaustion: “...*you know she feels she has to be by my side and the way to do that is to be an anorexic... it’s so, so draining...*” ^72^ (p. 1345). Many struggled to juggle personal, family, and work demands, often neglecting their own needs: “*...I feel bad to think of my own needs...*” ^72^ (p. 1343). This burden mainly fell on mothers, especially when fathers were less involved: “*you feel like you’re a single parent*” ^63^ (p. 1827).

Caregivers commonly experienced feeling powerless ^50^ ^,^ ^51^ ^,^ ^54^ ^,^ ^59^ ^,^ ^62^ ^,^ ^63^ ^,^ ^68^ ^,^ ^75^, describing it as “*going round in circles*” ^59^ (p. 495) or being “*boxed into a corner*” ^59^ (p. 497). This powerlessness could evolve into “*feelings of anger*” ^68^ (p. 5), particularly when the ill person resisted change, leading to perceptions of “*there’s no hope*” ^51^ (p. 396). Guilt was also pervasive, with some caregivers feeling responsible for the illness ^50^ ^,^ ^51^ ^,^ ^54^ ^,^ ^57^ ^,^ ^59^ ^,^ ^63^ ^,^ ^69^, describing it as their “*greatest failure*” ^63^ (p. 1827), regretting having “*missed*” ^63^ (p. 1827) something crucial, or believing they were “*not strong enough*” ^58^ (p. 496).

Another prevalent feeling was that of being forgotten or having no space for one’s own concerns, particularly among siblings ^62^ ^,^ ^65^ ^,^ ^66^ ^,^ ^67^ ^,^ ^70^ ^,^ ^71^ ^,^ ^75^. Some questioned their place in the family: “*do I exist in this world, really, or is it just her?*” ^62^ (p. 87); others felt guilty for needing support: “*...I felt so guilty for reaching out for help because I wasn’t the one who was struggling here, it was my sibling*” ^70^ (p. 4).

### Descriptive theme 3 - Challenges in accessing care: individual and social barriers

This theme reveals the social and personal challenges families face when coping with anorexia nervosa/bulimia nervosa which often intensify their suffering. Participants described pressure from a society that idealizes thinness, leading to intrusive body-related comments, even from family members ^55^ ^,^ ^56^ ^,^ ^69^ ^,^ ^75^. Discrimination and rejection by peers and relatives also emerged in their narratives, as one participant shared being referred to by family members as the “*evil and parasitic twin*” ^75^ (p. 8).

Parents struggled to recognize the eating disorder or perceiving its severity ^54^ ^,^ ^57^ ^,^ ^62^ ^,^ ^63^ ^,^ ^70^ ^,^ ^75^. Changes were seen as simply part of “*growing up and being a teenager*” ^54^ (p. 46), were obscured by their children’s lies, or even praised by the parents for fitting the thin ideal. Even when signs were noticed, a sense of confusion prevailed: “*when something is wrong in your house, you definitely know - but we didn’t know what*” ^63^ (p. 1824). Overwhelmed by uncertainty, parents became hypervigilant, seeking clarity wherever possible. As one mother stated: “*as I put all of the pieces together, I was talking about it to anybody and everybody who would listen, because I needed to know if you were seeing...*” ^63^ (p. 1828).

Moreover, complexity of the diagnosis alongside the stigmas and stereotypes surrounding mental disorders hinders its understanding. Participants reported facing barriers due to misconceptions that reduced eating disorders to eating issues ^50^ ^,^ ^52^ ^,^ ^54^ ^,^ ^55^ ^,^ ^59^ ^,^ ^60^ ^,^ ^61^ ^,^ ^62^ ^,^ ^63^ ^,^ ^66^ ^,^ ^68^ ^,^ ^69^ ^,^ ^71^ ^,^ ^72^ ^,^ ^75^. One mother’s account reflects the discomfort of being judged by others regarding how she should raise her daughter: “*everybody seemed to think the easiest solution was to sit her down with a meal*” ^54^ (p. 52).

Such limited understanding frequently resulted in misunderstandings that further distanced individuals (“*there’s always going to be a barrier that they’re not going to understand*” ^60^ [p. 37]) and families from support (“*you know people don’t seem to have much sympathy because they see it as a self-inflicted thing*” ^54^ [p. 52]). This difficulty in understanding anorexia nervosa/bulimia nervosa can foster denial, hinder recognition of severity, and delay treatment ^54^ ^,^ ^68^ ^,^ ^69^ ^,^ ^75^.

Eating disorders are an experience often surrounded by a silent secrecy, a secret that is often sustained by what is left unsaid yet still felt by others, creating an invisible barrier between the suffering individual and others ^49^ ^,^ ^50^ ^,^ ^52^ ^,^ ^53^ ^,^ ^57^ ^,^ ^58^ ^,^ ^61^ ^,^ ^62^ ^,^ ^63^ ^,^ ^68^ ^,^ ^71^ ^,^ ^75^. Participants cited several reasons for maintaining this secret: shame, protecting the family, and privacy. However, the outcome of this secret evidently results in a profound sense of loneliness: “*it’s very isolating*” ^63^ (p. 1826).

Studies indicate that resistance to treatment, by both individuals and families, can hinder progress ^50^ ^,^ ^51^ ^,^ ^54^ ^,^ ^61^ ^,^ ^62^ ^,^ ^65^ ^,^ ^70^ ^,^ ^75^. Some relatives resisted involvement, seeing it as “*strange*” ^62^ (p. 90) or unnecessary: “*...she is of legal age, so they should not refer to us*” ^65^ (p. 6). Others feared judgment from professionals: “*our parents felt any disclosures I made about ‘family issues’ would be a ‘betrayal’... drawing shame and judgment onto the family*” ^75^ (p. 15).

Numerous studies report serious shortcomings in family-related healthcare ^51^ ^,^ ^54^ ^,^ ^57^ ^,^ ^59^ ^,^ ^61^ ^,^ ^62^ ^,^ ^63^ ^,^ ^64^ ^,^ ^65^ ^,^ ^66^ ^,^ ^67^ ^,^ ^70^ ^,^ ^71^ ^,^ ^72^ ^,^ ^75^, including poor communication, long wait, and outdated practices like using body mass index (BMI) as the sole evaluation criterion ^51^ ^,^ ^52^ ^,^ ^57^ ^,^ ^61^ ^,^ ^63^ ^,^ ^64^ ^,^ ^65^ ^,^ ^70^ ^,^ ^71^. Services often overlooked families’ specific needs ^53^ ^,^ ^67^, with siblings feeling excluded and unsupported due to parent-focused approaches ^62^ ^,^ ^66^ ^,^ ^70^.

Professionals were often perceived as dismissing or minimizing the illness ^51^ ^,^ ^54^ ^,^ ^57^ ^,^ ^63^ ^,^ ^64^ ^,^ ^65^ ^,^ ^71^ ^,^ ^75^. One mother recalled a nutritionist saying “*she’s thin, but not unhealthy*” ^63^ (p. 1828) while another professional remarked: “*well, you’re not, you’re too heavy*” ^64^ (p. 4). Many parents felt they had to fight to be heard: “*...I am only listened to if I raise my voice and become strident*” ^65^ (p. 6). This struggle often leaves families overwhelmed as they try to fill the gaps left by the healthcare system, increasing their distress.

### Descriptive theme 4 - Pathways to coping: supporting the whole family - a public health issue

This theme explores how families cope with anorexia nervosa/bulimia nervosa, beginning with understanding and accepting the condition ^50^ ^,^ ^54^ ^,^ ^62^ ^,^ ^65^ ^,^ ^68^ ^,^ ^69^ ^,^ ^71^ ^,^ ^72^ ^,^ ^75^: “*till I put the name to it, I wasn’t strong enough to fight*” ^54^ (p. 50). This led to more open communication ^49^ ^,^ ^50^ ^,^ ^52^ ^,^ ^53^ ^,^ ^62^ ^,^ ^65^ ^,^ ^68^ ^,^ ^70^ ^,^ ^71^ ^,^ ^72^, fostering positive emotions and a sense of being “*less lonely*” ^68^ (p. 6).

As their understanding of eating disorders deepened, families expanded their caregiving beyond food-related concerns, adopting more holistic ways to support their loved one ^49^ ^,^ ^50^ ^,^ ^52^ ^,^ ^53^ ^,^ ^62^ ^,^ ^65^ ^,^ ^68^ ^,^ ^70^ ^,^ ^71^ ^,^ ^72^. Some offered emotional support through gestures like making “*a playlist for her on Spotify*” ^76^ (p. 997) or helping with transport costs to prevent weight loss from long walks. Others emphasized the importance of boundaries and responsibilities, recognizing that recovery also involves accountability ^66^.

Some studies highlighted the value of self-care and maintaining routine as key to coping and recovery ^51^ ^,^ ^52^ ^,^ ^62^ ^,^ ^65^ ^,^ ^70^ ^,^ ^72^. One mother shared that participating in treatment eased her anxiety: “*they made me feel like... it’s not just X there is actually me here somewhere if you look around*” ^51^ (p. 396). Distancing also emerged as a protective strategy, enabling some caregivers to sustain long-term support for their loved one ^49^ ^,^ ^50^ ^,^ ^62^ ^,^ ^65^ ^,^ ^66^ ^,^ ^68^ ^,^ ^72^ ^,^ ^76^: “*better to keep some distance and be able to stay the course*” ^66^ (p. 5).

Families’ hope that “*you’ve got to see light at the end of the tunnel*” ^67^ (p. 6) is what enables them to sustain their long-term efforts in supporting their relative’s recovery ^50^ ^,^ ^51^ ^,^ ^57^ ^,^ ^62^ ^,^ ^67^ ^,^ ^69^ ^,^ ^75^ ^,^ ^76^. Others emphasized the role of personal autonomy in recovery, noting the need for some independence from the family ^54^ ^,^ ^57^ ^,^ ^64^ ^,^ ^68^. As one sibling expressed: “*they have to break away... to feel they are independent in relation to the family*” ^68^ (p. 6).

Hope in recovery and trust in the individual’s responsibility were linked to emotional growth among family members ^49^ ^,^ ^57^ ^,^ ^62^ ^,^ ^72^ ^,^ ^75^. Many reported improved relationships with food and the body, along with greater self-awareness, responsibility, and recognition of personal strengths - “*learn to love and have kindness for the body I’m in now*” ^75^ (p. 14). In some cases, motherhood became a powerful incentive for change ^49^ ^,^ ^53^ ^,^ ^58^ ^,^ ^61^: “*I have to change something. I can’t have a girl and go on in the same line. So I said I would give treatment another shot*” ^58^ (p. 72).

Several studies underscore the importance of strengthening family relationships to face ongoing challenges ^50^ ^,^ ^54^ ^,^ ^60^ ^,^ ^62^ ^,^ ^63^ ^,^ ^65^ ^,^ ^71^ ^,^ ^73^ ^,^ ^74^ ^,^ ^76^. One mother shared: “*...the openness in our home has been a strength of ours*” ^71^ (p. 6). Support from both close relatives and broader care networks also proved essential ^49^ ^,^ ^50^ ^,^ ^55^ ^,^ ^56^ ^,^ ^61^ ^,^ ^62^ ^,^ ^64^ ^,^ ^68^ ^,^ ^70^ ^,^ ^75^ ^,^ ^76^, as another mother acknowledged: “*it is all thanks to their father, who is warm, loving, and supportive*” ^49^ (p. 45).

Support from healthcare services was vital not only for the individual’s treatment but also for caregivers ^50^ ^,^ ^51^ ^,^ ^57^ ^,^ ^61^ ^,^ ^62^ ^,^ ^64^ ^,^ ^68^ ^,^ ^70^ ^,^ ^75^ ^,^ ^76^, as one participant reflected: “*they were our carers as well as X’s carers*” ^51^ (p. 396). Many called for improvements in care addressing both patients and families ^51^ ^,^ ^52^ ^,^ ^57^ ^,^ ^64^ ^,^ ^65^ ^,^ ^67^ ^,^ ^68^ ^,^ ^70^. One participant stressed the importance of healthcare services acknowledging caregivers’ needs, stating: “*you can’t ignore the parent and the parent’s feelings...*” ^51^ (p. 397).

Based on these results, we further developed two analytical themes.

### Analytical theme 1 - Interplay between anorexia/bulimia nervosa and family relations: a complex relationship that feeds on itself

Our findings reveal a complex dynamic between anorexia/bulimia nervosa and the family system. One recurring pattern involves a symbiotic bond with the mother, marked by emotional enmeshment, blurred boundaries, and excessive dependency, whereas the father often appears distant or emotionally absent, resulting in an imbalance in parental roles. In this context, the eating disorders may reflect not only an individual pathology but also a manifestation of relational fragilities rooted in the family’s history: a means of expressing, resisting, or defending against the tensions and vulnerabilities embedded in the family dynamics.

Eating disorder affects both the individual and the entire family, generating emotional strain, caregiver burden, and persistent feelings of guilt, helplessness, and frustration. As the disorder progresses, family life often revolves around the affected member’s needs, leading to the neglect of other members - particularly siblings - and to disrupted routines. When it involves adult women, parental care is frequently overlooked and marital relationships become impoverished. Over time, conflicts intensify, communication deteriorates, and emotional bonds weaken, fostering isolation and loneliness across the family.

Consequently, the disorder not only alters family configuration, affecting each member of the group, but is also influenced by them, reflecting relational fragilities, unspoken tensions, and emotional vulnerabilities. Thus, understanding this complex interplay requires addressing both the individual symptoms and the underlying relational dynamics that both sustain and are sustained by the disorder.

### Analytical theme 2 - A comprehensive approach to care that embraces all family members

Addressing complex mental health conditions requires engaging the whole family, as these conditions affect both individuals and the broader family system. While individual treatment remains essential, attending to the family context is equally important, given that family dynamics often contribute to both eating disorder development and maintenance. Family members may experience significant emotional burdens as they cope with a loved one’s illness. Consequently, involving the whole family can provide crucial support and stability throughout the recovery process.

Families often face the stigma associated with mental illness which can lead to shame and a profound sense of isolation, exacerbated by limited support from society and healthcare providers. This stigma may also be internalized within the family, hindering help-seeking behaviors. A holistic approach must therefore extend beyond individual treatment or food- and body-related concerns, encompassing social contexts and prioritizing the well-being of all family members.

In this regard, primary health care plays a crucial role in family-centered mental health interventions. As the entry into the healthcare system, primary care is well positioned to identify early signs of distress, support families, and coordinate referrals. For this to be effective, primary care professionals must be adequately trained to recognize the multifaceted nature of eating disorders - not only in terms of symptomatology but also regarding their broader socioemotional impact on the entire family system. Strengthening primary care capacity to address these dimensions is therefore essential for fostering a more accessible, integrated, and empathetic care model-one that promotes the well-being of both individuals and their support network.

## Discussion

Our review synthesized qualitative evidence on families’ experiences with eating disorders, highlighting the emotional complexities and challenges involved. Analysis identified four descriptive themes, exploring the dynamics between the eating disorder and family organization, factors that exacerbate suffering, and coping strategies employed by family members.

Studies indicate a relational pattern in families affected by eating disorders in which preexisting relational fragilities become further intensified by disorder onset ^8^ ^,^ ^9^ ^,^ ^10^ ^,^ ^11^ ^,^ ^12^ ^,^ ^13^ ^,^ ^14^. Findings from our review corroborate this observation, revealing emotional enmeshment with the maternal figure ^49^ ^,^ ^51^ ^,^ ^55^ ^,^ ^56^ ^,^ ^60^ ^,^ ^69^ alongside distancing or emotional absence of the paternal figure ^55^ ^,^ ^56^ ^,^ ^60^ ^,^ ^69^ ^,^ ^73^ ^,^ ^74^ ^,^ ^76^. These dynamics manifest in ambivalent relational patterns, sometimes characterized by intense intrusion and conflict, and sometimes by emotional withdrawal and neglect.

An emotionally adverse environment can contribute to psychological vulnerability. In this context, symptoms may function as a defense mechanism against fragile family bonds ^56^ ^,^ ^60^ ^,^ ^72^ ^,^ ^73^ ^,^ ^75^. Marital strain can lead parents to unconsciously displace their conflicts onto the child’s illness ^55^ ^,^ ^60^ ^,^ ^69^, whereas children may use the disorder as a means of gaining control or escaping family tension. Thus, eating disorders symptoms emerge as a signal that something is wrong and requires change.

While the illness may serve as self-protection, it can also trap individuals in cycles of isolation, dependence, and suffering, reinforcing the very dynamics they sought to escape. This self-perpetuating interplay between eating disorders and the family system is explored in the first analytical theme, showing how they become mutually reinforcing over time. As the eating disorder progresses, the family becomes increasingly burdened by the ongoing strain of a chronic and enduring illness ^13^ ^,^ ^20^. Caregivers experience considerable distress ^21^ ^,^ ^22^ ^,^ ^23^ and the whole family becomes trapped in a cycle of suffering generated by the disorder, with daily routines and relationships progressively disrupted and shaped by its presence ^50^ ^,^ ^51^ ^,^ ^57^ ^,^ ^59^ ^,^ ^62^ ^,^ ^65^ ^,^ ^71^ ^,^ ^75^.

Given the complexity of the familial context, care must extend beyond the individual to encompass the whole family, a focus explored in the second analytical theme. Studies highlight the crucial role of family inclusion in treatment ^27^ ^,^ ^28^ ^,^ ^31^ ^,^ ^32^, recognizing it as a key ally in both recovery and overall improvement of the affected individual’s mental health. Review findings indicate that such inclusion is highly beneficial, as strategies adopted not only by the individual but also by family members can significantly contribute to the recovery process ^49^ ^,^ ^50^ ^,^ ^52^ ^,^ ^53^ ^,^ ^62^ ^,^ ^65^ ^,^ ^68^ ^,^ ^70^ ^,^ ^72^.

Although the family constitutes a cornerstone of contemporary mental health care models, evidence in the specialized literature remains poor on how to develop comprehensive care that meaningfully integrates both individuals and their families ^24^. Consequently, families often face numerous barriers, challenges and unmet needs when seeking treatment, particularly due to lack of professional support ^51^ ^,^ ^54^ ^,^ ^57^ ^,^ ^59^ ^,^ ^61^ ^,^ ^67^ ^,^ ^70^ ^,^ ^72^ ^,^ ^75^. Despite many studies reporting the strategies that participants employ to cope with eating disorders and care for themselves ^51^ ^,^ ^52^ ^,^ ^62^ ^,^ ^65^ ^,^ ^70^ ^,^ ^72^, there remains an urgent need for systemic improvement to establish care pathways that are more closely aligned with the guiding principles of mental health policy, aiming to promote more supportive, inclusive, and resilient eating disorder treatment ^26^.

Regarding study limitations, methodological quality analysis identified deficiencies in the reflexivity criterion. Specifically, several studies did not adequately address the researcher’s stance or the potential influence they may have exerted in the research context. No study specifically addressed topics such as socioeconomic conditions and domestic violence, which can exacerbate conflicts that weaken family bonds. Evidence shows that families living in poverty often face multiple stressors that affect emotional well-being and greater challenges in accessing healthcare and social support. These limitations underscore the need for further research that more fully captures the complexity of the phenomenon under investigation.

## Conclusion

Our meta-synthesis produced deeper understanding of family dynamics around eating disorders. By revisiting previous research findings, this study reinterpreted the results of primary qualitative studies on family’s perspective regarding changes in family relations following a member’s diagnose of anorexia nervosa and bulimia nervosa. Interpretive description produced knowledge with direct implications for improving practices and policies as global policies emphasize the need for tailored interventions to reduce eating disorder harms among vulnerable people. Understanding the risk behaviors associated with eating disorders may inform public health policies aimed at developing suitable programs and interventions.

Particularly, it enabled exploring the bidirectional dynamics between family functioning and eating disorders using a rigorous methodology to identify patterns and recurring themes across different qualitative studies and gain a more in-depth, systematic understanding of the existing evidence in the literature. Further, the study considered the unique and singular perspective of the family which is a key element in the recovery process.

In considering the complex relation between anorexia nervosa/bulimia nervosa and family dynamics, along with their reciprocal influences, our findings can help offer a more integrated and comprehensive to this population. This perspective aligns with psychosocial care principles which emphasize engaging the family in all its complexity. Thus, including the family in psychosocial care not only acknowledges its potential as a source of support for the individual experiencing psychological distress, but also recognizes the family’s own need for adequate support to cope with physical, emotional, and psychological burden. Ultimately, this approach facilitates reorganizing and strengthening the family unit ^77^.

## Data Availability

The sources of information used in the study are indicated in the body of the article.
