# Supplementary material for: Family member’s perceived impact of anorexia nervosa and bulimia nervosa on family dynamics: a qualitative systematic review and meta-synthesis
Source: Cad Saude Publica. 2026 May 1;42:e00181525. doi: 10.1590/0102-311XEN181525 (PMC13143282; doi:10.1590/0102-311XEN181525)
Supplement: Supplementary Material [file 1678-4464-csp-42-EN181525-s.pdf]

SUPPLEMENTARY MATERIAL

Box S1 Example of application of the SPIDER strategy in Scopus database search.

**S**<br>*Sample*	Eating Disorder OR Eating Disorders OR Feeding Disorders OR Feeding and Eating Disorders OR Eating and Feeding Disorders OR Appetite Disorder OR Appetite Disorders OR Anorexia Nervosa OR Anorexia OR Bulimia Nervosa OR Bulimia
**Pi**<br>*Phenomenon of Interest*	Family Dynamic OR Family Dynamics OR Family Relation OR Family Relations OR Family Relationship OR Family Relationships OR Family Functioning OR Family Interaction OR Family Interactions OR Intergenerational Relation OR Intergenerational Relations OR Parent-Child Relation OR Parent-Child Relations OR Parent-Offspring Interaction OR Parent-Offspring Interactions OR Mother-Child Relation OR Mother-Child Relations OR Mother-Child Interaction OR Mother-Child Interactions OR Mother-Child Relationship OR Mother-Child Relationships OR Sibling Relation OR Sibling Relations OR Father-Child Relation OR Father-Child Relations OR Father-Child Relationship OR Father-Child Relationships OR Parenting OR Carer's experiences
**D**<br>*Design*	Focus Group OR Focus Groups OR Phenomenology OR Ethnography OR Anthropology OR Ethnographic research OR phenomenological research OR Group Interview OR Group Interviews OR Grounded Theory OR Interviewers OR Interviewer OR Psychologic Interview OR Psychologic Interviews OR Psychological Interview OR Psychological Interviews
**E**<br>*Evaluation*	Perception OR Experience OR Meaning OR Perspective OR Life Experience OR Life Experiences OR Life Course Perspective OR Life Course Perspectives
**R**<br>*Research type*	Qualitative Research OR Research Qualitative OR Qualitative studies OR Qualitative OR Empirical Research OR Research Empirical

Box S2 References that were excluded after their full text was reviewed and the reasons for exclusion.

**Excluded references**	**Reasons**
Buser, J. K., Parkins, R. A., Gelin, S., Buser, T., & Kearney, A. (2016). Relationships with individuals facing eating disorder symptoms: Using transcendental phenomenology to understand this experience. The Family Journal, 24(4), 325–334. https://doi.org/10.1177/1066480716663204	This study aimed to explore the experience of friends, nonparental family members and romantic dating partners of people with an eating disorder. However, the results section did not present information regarding the impact of eating disorders on the dynamics of family relationships. Thus, constituting the wrong phenomenon of interest.
Taborelli, E., Easter, A., Keefe, R., Schmidt, U., Treasure, J., & Micali, N. (2016). Transition to motherhood in women with eating disorders: A qualitative study. Psychology and Psychotherapy, 89(3), 308–323. https://doi.org/10.1111/papt.12076	This study aimed to explore the pregnancy and motherhood experiences of women with eating disorders, focusing on their personal experiences and their relationship with their changing bodies. However, the results highlighted the impact of pregnancy on these women's lives, including its effect on ED symptoms. Thus, constituting the wrong phenomenon of interest.
Castañeda, L. A. R., Palos, P. A., Heredia, M. E. R., Santoncini, C. U. (2018) Parents' and daughters' perception of family aspects associated with the onset of an eating disorder. Revista Mexicana de Trastornos Alimentarios. 9(1): 71-81. https://doi.org/10.22201/fesi.20071523e.2018.1.484	This study aimed to identify and understand the family factors related to the development of eating disorders, based on the perspectives of parents of daughters with ED and women who are affected by it. However, the results focus on the etiology of the eating disorder, without addressing changes in family relationships after the onset of ED. Thus, constituting the wrong phenomenon of interest.
Lyons, G., McAndrew, S., & Warne, T. (2019). Disappearing in a female world: men's experiences of having an eating disorder (ED) and how it impacts their lives. Issues in Mental Health Nursing, 40(7), 557–566. https://doi.org/10.1080/01612840.2019.1576815	This study investigated the lived experiences of men diagnosed with an ED and how it affects various aspects of their daily lives. However, the results section did not present information about the impact of the eating disorder on the participants' family dynamics. Thus, constituting the wrong phenomenon of interest.
Reid M, Wilson-Walsh R, Cartwright L, Hammersley R. (2020). Stuffing down feelings: Bereavement, anxiety and emotional detachment in the life stories of people with eating disorders. Health & Social Care in the Community. 28: 979–987. https://doi.org/10.1111/hsc.12930	This study aimed to explore the life stories of individuals with eating disorders to have a deeper understanding of the potential factors contributing to their development. However, the study did not specify the participants' diagnoses, which constituted a wrong sample.
Vu-Augier de Montgrémier, M., Moro, M. R., Chen, J., Blanchet, C., & Lachal, J. (2020). Eating disorders and representations of the role of women in China: A qualitative study. European Eating Disorders Review, 28(2), 211–222. https://doi.org/10.1002/erv.2717	This study investigated the representations of femininity and the expectations related to the female gender among Chinese girls with eating disorders and their parents. However, the results section did not provide information on the impact of the eating disorder on the participants' family dynamics, thereby misrepresenting the phenomenon of interest.
Dahill, L. M., Morrison, N. M. V., Touyz, S., Mitchison, D., Bussey, K., Mannan, H., & Hay, P. (2023). An exploration of how adolescents experience and reason their parents' comments on their weight, shape, and eating. Journal of Adolescence, 95(7), 1488–1504. https://doi.org/10.1002/jad.12221	This study aimed to investigate adolescents' experiences with parental communication regarding body weight, shape, and eating. However, the participants' profiles did not mention an eating disorder diagnosis. Thus, constituting the wrong sample.
Eaton, C. M. (2023). Infant feeding experiences of women who recovered from anorexia nervosa. Journal of Obstetric, Gynecologic & Neonatal Nursing, 52(5), 384-393. https://doi.org/10.1016/j.jogn.2023.06.005	This study aimed to describe the infant feeding experiences of women who recovered from anorexia nervosa However, access to the article could not be obtained. The full search process for the article was as follows: 1) Attempted access via the website https://www.jognn.org/article/S0884-2175(23)00211-3/abstract through institutional access; 2) Attempted access via the CAPES Periodicals Portal, using CaFE access; 3) Attempted access through the physical and digital library of the institution affiliated with the article's authors; 4) Direct email contact with the article's author; 5)Inquiry through the authors' network of researchers from other institutions to see if they had access to the article.
Korczynski, S., Khiar Zerrouk, A., Revah-Levy, A., Sibeoni, J., & Lachal, J. (2023). Issues around food in mixed families of adolescent girls with bulimia nervosa: A qualitative study with photo-elicitation. L'Encephale, 49(6), 606–611. https://doi.org/10.1016/j.encep.2022.08.019	This study aimed to find out about the role of food in family relationships within the specific context of mixed families with an adolescent suffering from bulimia nervosa. However, the results highlighted the cultural context of mixed families, not addressing the impact of ED on family dynamics. Thus, constituting the wrong phenomenon of interest.

Source: prepared by the authors.
